# Association between glucocorticoid administration and outcomes in patients with ARDS based on the MIMIC-III database

**DOI:** 10.1097/MD.0000000000039239

**Published:** 2024-08-09

**Authors:** Zhonghua Lu, Yan Tang, Mei Liu, Lijun Cao, Hu Chen, WeiLi Yu, Yun Sun

**Affiliations:** a The First Department of Critical Care Medicine, The Second Affiliated Hospital of Anhui Medical University, Hefei, Anhui Province, China; b Department of Rehabilitation and Rehabilitation Medicine, The Second Affiliated Hospital of Anhui Medical University, Hefei, Anhui Province 230601, China.

**Keywords:** ARDS, glucocorticoid, intensive care unit, mortality

## Abstract

This study aimed to investigate the association between glucocorticoid administration and outcomes in critically ill patients with ARDS using the Medical Information Mart for Intensive Care (MIMIC)-III database. Data were collected from the MIMIC-III database, which consists of critically ill participants between 2001 and 2012 in the USA. A total of 1831 adult critically ill patients with ARDS were enrolled from the MIMIC-III database. The 60-day and in-hospital mortality, were the primary endpoints. Secondary outcomes included length of stay (LOS) in the hospital and intensive care unit (ICU), 28-day ventilator-free days, ICU mortality, and 28-day mortality. A total of 1831 patients were included in the data analysis. After propensity score (PS) matching, 464 patients diagnosed with ARDS were matched between the glucocorticoid treatment and control groups. Glucocorticoids were associated with increased in-hospital mortality [hazard ratio (HR) 1.32; 95% CI 1.01–1.71; *P* = .039], longer ICU stay [HR 2.25; 95% CI 0.84–3.65; *P =* .002], and shorter ventilation-free days at 28 days in all ARDS patients [HR −2.70; 95% CI −4.28–-1.13; *P* = .001]. The 60-day mortality was higher in the glucocorticoid group (44.83% vs 35.34%; *P* = .154; HR 1.24; 95% CI 0.93–1.66). Excluding the impact of the glucocorticoid initiation time, from day 15 to day 60, mortality was significantly higher in the glucocorticoid group compared to the non-glucocorticoid group (27.16% vs 12.70%; *P* < .001; HR 1.75; 95% CI 1.32–2.32). Glucocorticoid administration was associated with worse 60-day and in-hospital survival, longer ICU stay, and shorter ventilator-free days on day 28 in patients with ARDS. Our findings suggest careful consideration of glucocorticoids for ARDS.

## 1. Introduction

Acute respiratory distress syndrome (ARDS) is a common and clinically complex acute pulmonary inflammatory syndrome with a mortality rate of up to 40%.^[1]^ The pathological changes of ARDS include exudative, proliferative, and fibrotic phases.^[2–4]^ Its persistent inflammation, parenchymal cell proliferation, and collagen deposition may respond to anti-inflammatory corticosteroids, making them a potentially beneficial treatment option.^[5,6]^ However, previous randomized trials failed to provide convincing evidence of consistent results in favor of corticosteroid therapy^.[7–9]^ They demonstrated that corticosteroids have advantages over controls in reducing ARDS mortality, the duration of mechanical ventilation, and the length of hospital stay. The MIMIC-III database includes real-world clinical data, reflecting the diversity of actual clinical practice, and offers broad sample coverage with extensive patient data, making it advantageous for rare disease and subgroup analyses. The 60-day mortality rate reflects the mid-term effects of glucocorticoid treatment, including delayed complications. Therefore, this study hypothesizes that glucocorticoids can significantly reduce the 60-day mortality in ARDS patients and aims to assess their impact on 60-day mortality and other prognostic indicators. Despite existing studies, data remain insufficient.

Published clinical studies or meta-analyses on corticosteroid therapy for ARDS have reported inconsistent conclusions, which may be due to a variety of reasons.^[10]^ The different choices of follow-up time and heterogeneity of etiology in ARDS patients contributed to the inconsistent results of previous studies.^[9–11]^ The therapeutic efficacy of glucocorticoids is evident in the initial phases, while infections and neuromuscular dysfunction manifest in the later stages. Therefore, short-term outcomes as study endpoints may underestimate the risks of glucocorticoid therapy. In addition, clinical outcomes were also associated with the presence of hypertension, cardiovascular disease, and other underlying diseases, immune impairment, glucocorticoid dose, and time from onset to glucocorticoid initiation.^[7,12–14]^ We conducted a retrospective study of ARDS patients using Medical Information Mart for Intensive Care III (MIMIC-III) data to identify subgroups that could benefit from glucocorticoid therapy.

## 2. Methods

### 2.1. Study design and data sources

This was a retrospective cohort study of 1831 patients with ARDS according to the Berlin definition. We obtained all data from the MIMIC-III, a single-center, freely accessible database containing more than 60,000 health-related hospitalizations from 2001 to 2012 in the Intensive Care Unit (ICU) of the Beth Israel Deaconess Medical Center in Boston. We completed the online course and passed online exams (no. 43881398) to gain access to the database. The establishment of the MIMIC-III database was approved by the Institutional Review Board of the Beth Israel Deacons Medical Center and the Massachusetts Institute of Technology. Because the hospitalization information was anonymous, informed consent was not required.

## 3. Data collection and definitions

All the patients were screened in the database. Our inclusion criteria were as follows: at least 18 years, ICU stay more than 72 hours, and an ARDS diagnosis meeting the Berlin standard at the time of ICU admission. The Berlin standard included acute onset, arterial oxygen partial pressure (PaO2)/fraction of inspired oxygen (FiO2) < 300 mm Hg, positive end-expiratory pressure (PEEP) ≥ 5 cmH_2_O on the first day of ICU admission, bilateral infiltrates on chest radiograph, and absence of heart failure. According to the Berlin standard, ARDS was classified as mild (>200 mm Hg, ≤300 mm Hg), moderate (>100 mm Hg, ≤ 200 mm Hg), or severe (<100 mm Hg) based on the PaO2/FiO2 ratio.

We used a Structured Query Language to extract data. We extracted or calculated the following variables, including baseline characteristics (age, sex, ethnicity, admission type), the patients’ comorbidities, the Elixhauser Comorbidity Index (SID30), the risk factors leading to ARDS, vital signs within 24 hours after ICU admission (heart rate, temperature, mean arterial pressure, peripheral capillary oxygen saturation), severity of organ dysfunction (Simplified Acute Physiology Score II); Oxford Acute Severity of Illness Score (OASIS); Sequential Organ Failure Assessment (SOFA), PaO2/FiO2 at diagnosis, PEEP at diagnosis, and the treatments received (ventilation received; vasopressor therapy; renal replacement therapy; corticosteroid therapy).

## 4. Endpoints

The primary endpoints were in-hospital mortality and 60-day mortality. The 60-day mortality endpoint was selected to assess the mid-term effects of glucocorticoid therapy, including delayed complications and long-term benefits. This timeframe allows for evaluating the therapy’s effectiveness beyond the acute phase. Studies, such as Steinberg et al,^[7]^ indicate that the lasting impacts on mortality and morbidity are best captured at 60 days. Given the inclusion criteria of using glucocorticoids within 2 weeks, we evaluated 0–14 day and 15–60 day mortality rates to exclude the impact of inconsistent early usage on prognosis. The length of stay (LOS) in the hospital and ICU, 28-day ventilator-free days, ICU mortality, and 28-day mortality were considered secondary outcomes.

## 5. Statistical analysis

All data were processed and analyzed using STATA software. We presented the continuous variables as the median (interquartile range) and the differences between groups, and compared them using the Mann–Whitney test (for non-normal distributions), while categorical variables were presented as percentages and compared using the chi-square test. A 2-tailed *P* value < .05 was considered statistically significant.

To estimate the association between glucocorticoid administration and outcomes among patients with ARDS, propensity score matching was performed using greedy nearest-neighbor matching with a caliper of 0.2 standard deviations of the logit of the estimated propensity score. Patients were matched in a 1:1 ratio, such that each patient who was treated with glucocorticoid within 14 days after ICU admission was matched to a patient without glucocorticoid treatment. The standardized mean difference (SMD) was calculated to evaluate the efficiency of propensity score matching (PSM) in reducing differences between the 2 groups.

A Cox regression model was used to estimate the relationships between glucocorticoid administration and mortality outcomes, adjusting for confounding variables selected based on a *P* value < .05, in univariate analysis and potential confounders judged by the clinical expertise of our team. Similar to other studies,^[15]^ the selection of specific confounding variables was based on factors that are clinically and biologically considered to significantly impact patient prognosis, such as age, gender, underlying diseases, severity of illness, and other key treatments. Among these factors, those that showed statistical differences in the initial comparison were selected. Linear regression was used to assess the association between glucocorticoid use and the length of hospital/ICU stay and 28-day ventilator-free days.

Stratification analysis was conducted to explore whether the association between glucocorticoid administration and 60-day and in-hospital mortality differed across various subgroups classified by age, gender, emergency, ethnicity, diabetes, cardiopulmonary status, CRRT, SIRS, severity of ARDS, SOFA, moderate anemia, NLR (neutrophil lymphocyte ratio), or use of vasoactive medications. The analysis was performed on the population after PSM matching.

## 6. Patient and public involvement

Patients and/or the public were not directly involved in this study.

## 7. Results

### 7.1. Basic characteristics

**Figure 1.**
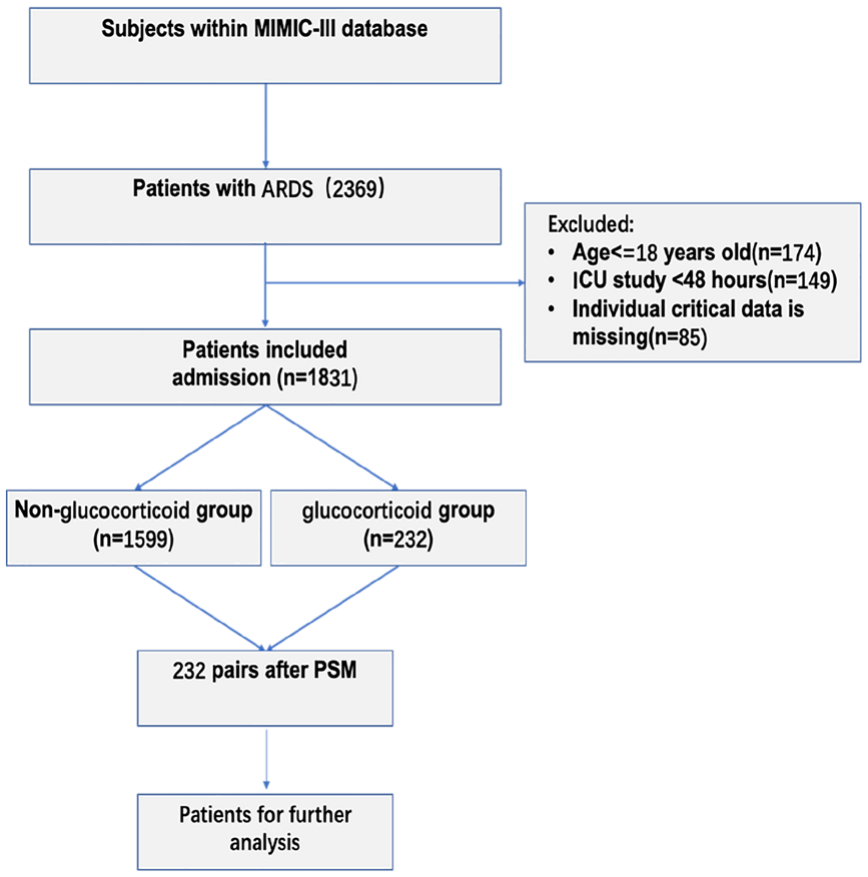
Flowchart of included patients. ARDS = acute respiratory distress syndrome, ICU = intensive care unit, MIMIC-III = Medical Information Mart for Intensive Care III, PSM = propensity score matching.

During the study period, 2369 patients were admitted for ARDS. After applying the exclusion criteria, 1831 eligible patients were enrolled. The exclusion criteria included the following: patients aged 18 years or younger (n = 174), those with an ICU stay of <48 hours (n = 149), and those with missing critical individual data (n = 85). Among the enrolled patients, 232 were exposed to glucocorticoids within 14 days of ARDS diagnosis, while 1599 ARDS patients did not meet the inclusion criteria (Fig. 1).

As shown in Table 1, there were significant differences between the glucocorticoid and non-glucocorticoid groups in several severity scales and treatments. Specifically, the glucocorticoid group had higher Simplified Acute Physiology Score II (43.00 [33.00–53.00] vs 40.00 [31.00–50.00], *P* = .037), SOFA (7.00 [5.00–10.00] vs 6.00 [3.00–9.00], *P* = .002), OASIS (40.00 [34.00–45.50] vs 38.00 [32.00–43.00], *P* = .005), and PaO2/FiO2 at diagnosis (112.29 [79.50–168.00] vs 133.33 [89.00–187.50], *P* = .001). More patients in the glucocorticoid group had moderate to severe ARDS (89.66% vs 80.30%, *P* = .001) and received vasoactive medication therapy (44.83% vs 31.08%, *P* < .001). Although the proportions of diabetic patients, female patients, and Caucasian patients in the glucocorticoid group were higher, these differences were not statistically significant compared to the control group.

**Table 1:** Basic characteristics of cohorts of patients treated with and without glucocorticoids.

Characteristic	Non-GC, n = 1599	GC, n = 232	*P*
Age (yr)	63.84 (50.38, 76.43)	62.56 (51.02, 76.27)	.815
Female	642 (40.16%)	107 (45.71%)	.084
Weight	80.00 (67.60, 95.60)	78.05 (67.00, 90.85)	.274
Height	172.72 (165.10, 182.88)	172.72 (162.56, 182.88)	.053
Emergency	1387 (86.82%)	204 (87.35%)	.616
Ethnicity (White)	1247 (77.99%)	168 (72.41%)	.058
SIRS	3.00 (3.00, 4.00)	3.00 (3.00, 4.00)	.958
WBC__Max_	14.60 (10.30, 19.90)	14.05 (10.25, 20.05)	.778
Plt__Min_	176.00 (111.00, 251.00)	178.50 (95.00, 270.00)	.919
Hemoglobin	9.40 (8.20, 10.90)	9.40 (8.40, 10.90)	.617
Bun__Max_	25.00 (16.00, 44.00)	26.50 (18.00, 41.50)	.233
Cr__Max_	1.20 (0.90, 1.90)	1.30 (0.90, 2.10)	.275
Lac__Max_	3.10 (1.80, 7.00)	2.80 (1.80, 6.10)	.610
NLR at diagnosis	10.34 (5.66, 15.40)	11.26 (5.14, 20.38)	.371
Smoking	1147 (71.56%)	169 (73.88%)	.725
Severity scale			
SAPSII	40.00 (31.00, 50.00)	43.00 (33.00, 53.00)	.037
OASIS	38.00 (32.00, 43.00)	40.00 (34.00, 45.50)	.005
SOFA	6.00 (3.00, 9.00)	7.00 (5.00, 10.00)	.002
LODS	6.00 (4.00, 8.00)	6.00 (4.00, 9.00)	.098
Coexisting disorder	930 (58.16%)	148 (63.79%)	.103
Cardiopulmonary	468 (29.27%)	67 (28.88%)	.903
Hypertension	531 (33.21%)	78 (33.62%)	.901
COPD	52 (3.22%)	9 (4.08%)	.619
Coronary	348 (21.76%)	43 (18.53%)	.262
Diabetes	462 (28.89%)	81 (34.91%)	.061
SID30	14.00 (5.00, 23.00)	14.50 (7.00, 25.00)	.125
PaO2/FiO2 at diagnosis	133.33 (89.00, 187.50)	112.29 (79.50, 168.00)	.001
PEEP	5.00 (5.00, 10.00)	5.00 (5.00, 10.00)	.732
Moderate-severe ARDS	1284 (80.30%)	208 (89.66%)	.001
CRRT	138 (8.63%)	29 (12.50%)	.056
Vasoactive treatment	497 (31.08%)	104 (44.83%)	<.001

Max or Min is the maximum or minimum value on the day of ARDS diagnosis. Data are presented as number (%) or median [interquartile range (IQR)].

ARDS = acute respiratory distress syndrome, BUN = blood urea nitrogen, COPD = chronic obstructive pulmonary disease, Cr = creatinine, CRRT = continuous renal replacement therapy, FiO2 = fraction of inspired oxygen, GC = glucocorticoids, Lac = lactate, LODS = logistic organ dysfunction score, NLR = neutrophil-to-lymphocyte ratio, PaO2 = partial pressure of oxygen, PEEP = positive end-expiratory pressure, Plt = platelet count, SAPSII = Simplified Acute Physiology Score II, SID30 = Elixhauser Comorbidity Index, SIRS = systemic inflammatory response syndrome, SOFA = sequential organ failure assessment, WBC = white blood cell count.

## 8. Relationship between glucocorticoid and outcomes

Cox proportional hazards (PH) models examined the differences in mortality outcomes between the 2 groups, and glucocorticoid use increased mortality at 60 days in a prematched cohort (HR, 1.26; 95% CI 1.02–1.56; *P* = .033) after adjusting for possible confounding factors associated with mortality (Table 2 and Table S1, http://links.lww.com/MD/N318). The proportional hazards assumption test based on Schoenfeld residuals is the premise of Cox regression, but the PH test results do not meet the hypothesis, and the -ln [-ln (survival)] graph also shows that there is crossover between the 2 groups (Fig. S1, http://links.lww.com/MD/N318).

**Table 2:** Outcomes of the patients with ARDS treated with or without glucocorticoids.

Outcome	Non-GC	GC	*P* value	HR	Lower 95% CI	Upper 95% CI
Prematched cohort	n = 1599	n = 232				
60-day mortality†	481 (30.08%)	104 (44.83%)	.033	1.26	1.02	1.56
0–14-day mortality†	251 (15.70%)	41 (17.67%)	.271	0.83	0.59	1.16
15–60-day mortality†	203 (12.70%)	63 (27.16%)	<.001	1.75	1.32	2.32
In-hospital mortality	665 (41.59%)	130 (56.03%)	.007	1.30	1.07	1.57
LOS in ICU*	8.88 (4.95, 16.16)	12.06 (7.57, 18.89)	.002	2.25	0.84	3.65
LOS in hospital*	16.24 (9.64, 25.72)	18.09 (12.89, 26.69)	.069	2.14	−0.17	4.45
ventilator-free days 28*	21.35 (0, 26.49)	14.10 (0, 23.78)	.001	−2.70	−4.28	−1.13
ICU mortality†	377 (23.58%)	84 (36.21%)	.076	1.24	0.98	1.58
28-day mortality†	376 (23.51%)	82 (35.34%)	.202	1.17	0.92	1.49
Matched cohort	n = 232	232				
60-day mortality†	82 (35.34%)	104 (44.83%)	.154	1.24	0.93	1.66
0–14-day mortality†	40 (17.24%)	41 (17.67%)	.643	0.90	0.58	1.40
15–60-day mortality†	42 (18.10%)	63 (27.16%)	.025	1.57	1.06	2.33
In-hospital mortality†	106 (45.69%)	130 (56.03%)	.039	1.32	1.01	1.71
LOS in ICU*	9.01 (5.38, 16.02)	12.06 (7.57, 18.89)	.003	2.70	0.90	4.50
LOS in hospital*	16.41 (9.78, 25.09)	18.09 (12.89, 26.69)	.112	2.51	−0.58	5.61
28-day VFDs*	21.04 (0, 26.35)	14.10 (0, 23.78)	.010	−2.78	−4.90	−0.67
ICU mortality†	69 (21.43%)	84 (36.21%)	.254	1.21	0.87	1.66
28-day mortality†	64 (27.59%)	82 (35.34%)	.344	1.17	0.84	1.63

Data are presented as number (%) or median [interquartile range (IQR)].

ARDS = acute respiratory distress syndrome, BUN = blood urea nitrogen, COPD = chronic obstructive pulmonary disease, Cr = creatinine, CRRT = continuous renal replacement therapy, FiO2 = fraction of inspired oxygen, GC = glucocorticoids, Lac = lactate, LODS = logistic organ dysfunction score, LOS = length of stay, NLR = neutrophil-to-lymphocyte ratio, PaO2 = partial pressure of oxygen, PEEP = positive end-expiratory pressure, Plt = platelet count, SAPSII = Simplified Acute Physiology Score II, SID30 = Elixhauser Comorbidity Index, SIRS = systemic inflammatory response syndrome, SOFA = sequential organ failure assessment, VFDs = ventilator-free days, WBC = white blood cell count.

* Linear regression was used to evaluate the association between glucocorticoid use and length of stay or ventilator-free days at day 28.

† Cox regression was used for estimating the impact of glucocorticoid use on mortality outcomes adjusting for confounding variables selected based on *P* value < .05 in univariate analysis.

Considering the interaction between treatment and time, the Kaplan–Meier survival curves indicated a significant change around day 14 for both patient groups. Since the inclusion criteria required patients to have used glucocorticoids within the previous 2 weeks, we stratified the mortality analysis into 2 periods: within the first 14 days and from day 15 to day 60. This stratification helps eliminate the influence of inconsistent glucocorticoid use duration within the initial 2 weeks and distinguishes the immediate effects of glucocorticoids from their longer-term effects, providing a clearer picture of how treatment timing influences patient outcomes. The PH assumption was retested for both intervals, with all *P* values >.05, confirming the assumption. The -ln[-ln(survival)] plot (Figs. S2–S3, http://links.lww.com/MD/N318) also showed parallel lines for the 2 groups, supporting the stratified analysis. The results revealed no significant difference in mortality within the first 14 days between the glucocorticoid and non-glucocorticoid groups [17.67% vs 15.70%, *P* = .271; hazard ratio (HR) 0.83, 95% confidence interval (CI) 0.59–1.16], suggesting that glucocorticoid treatment does not significantly impact early mortality within the acute phase of ARDS. However, from day 15 to day 60, mortality was significantly higher in the glucocorticoid group compared to the non-glucocorticoid group (27.16% vs 12.70%, *P* < .001; HR 1.75, 95% CI 1.32–2.32), indicating that glucocorticoid treatment is associated with increased mortality in the longer-term phase, even when accounting for variations in the initial timing of glucocorticoid therapy. Overall, the 60-day mortality was significantly higher in the glucocorticoid group (44.83% vs 35.34%, *P = *.154; HR 1.24, 95% CI 0.93–1.66; Table 2).

Hospital mortality was also higher in the glucocorticoid group than in the control group after adjustment for confounders (HR, 1.30; 95% CI 1.07–1.57; *P = *.007). Cox proportional hazards models were also used to compare the secondary endpoints, 28-day mortality, and ICU mortality, which were slightly higher in the glucocorticoid group but not statistically different from the control group (all *P* > .05; Table 2). Linear regression controlling for possible intervention factors showed that the total length of hospital stay and length of ICU stay were significantly longer in the glucocorticoid-treated group than in the control group (*P < *.05), while the 28-day ventilator-free days (VFDs) were shorter in the glucocorticoid-treated group (*P < *.05; Table 2).

**Figure 2.**
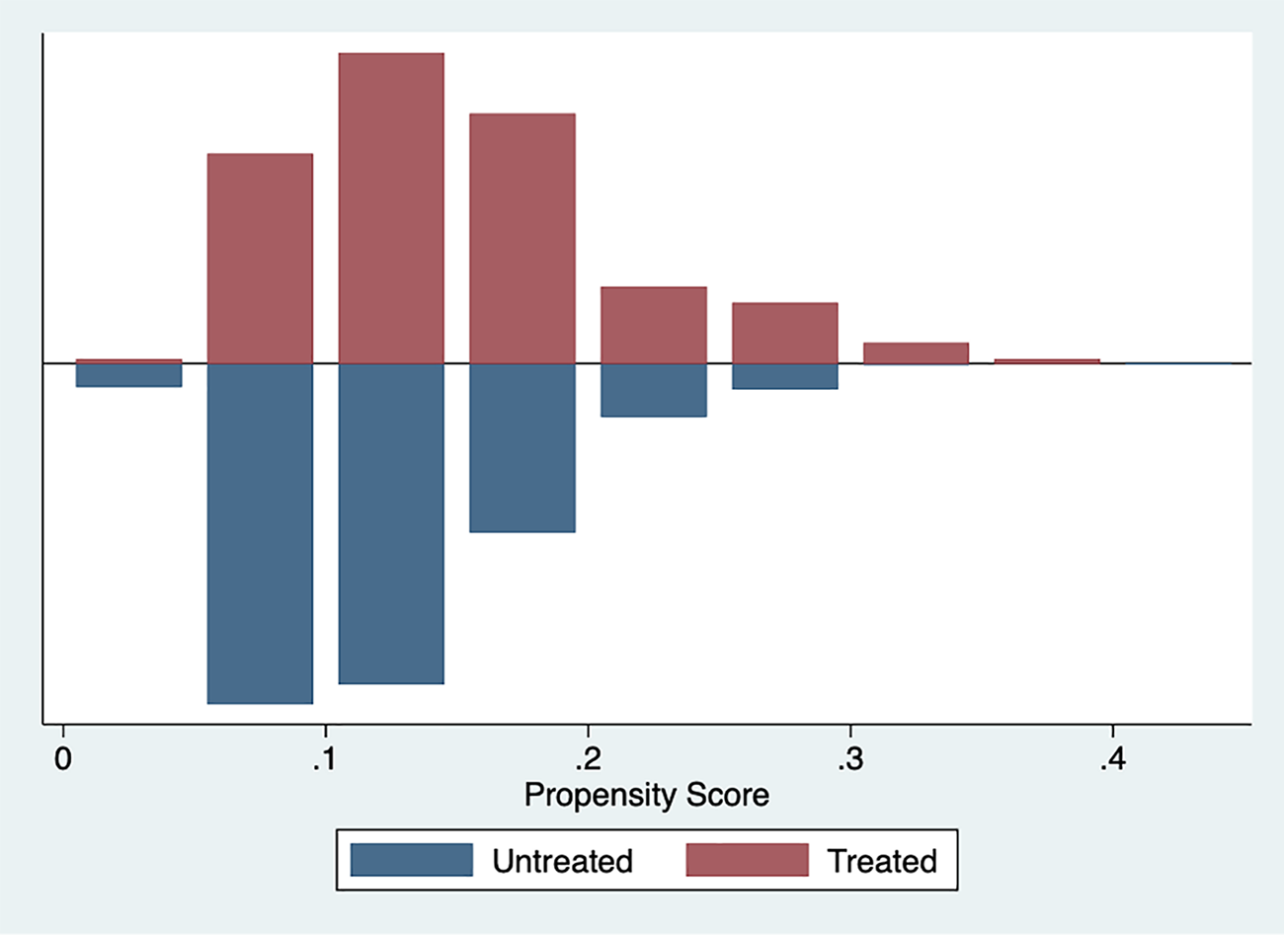
Graph shows the equilibrium before and after variable matching based on 60-day mortality.

**Figure 3.**
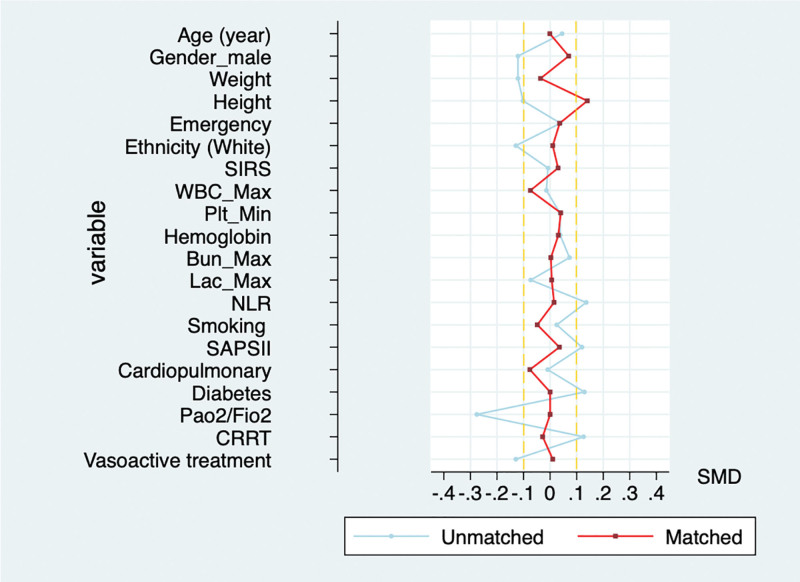
Standardized mean difference (SMD) of variables before and after propensity score matching. BUN = blood urea nitrogen, Lac = lactate, CRRT = continuous renal replacement therapy, FiO2 = fraction of inspired oxygen, NLR = neutrophil-to-lymphocyte ratio, PaO2 = partial pressure of oxygen, Plt = platelet count, SAPSII = Simplified Acute Physiology Score II, SIRS = systemic inflammatory response syndrome, SMD = standardized mean difference, WBC = white blood cell count.

In the PSM, 232 patients receiving glucocorticoid therapy based on 60-day mortality were matched with patients who did not receive glucocorticoid therapy in this study (Fig. 2: histogram of matching results). In Table 2, the matched patient characteristics are compared, and the SMD for all individual covariates are provided. After matching, the baseline profiles were well balanced between the 2 groups, with SMDs <10% for almost all variables (Table 2, Fig. 3, and Table S2, http://links.lww.com/MD/N318). Contrary to the prematched model results, glucocorticoids were not associated with a statistically significant increase in 60-day mortality, and the PH test results also met the hypothesis (*P* = .6564). Similar to the results in the prematched model, glucocorticoids increased in-hospital mortality (HR, 1.30; 95% confidence interval 1.07–1.57; *P =* .039) and length of ICU stay and reduced 28-day VFDs, but the effect on total length of stay was not statistically different after matching compared with the control group (Table 2).

## 9. Subgroup analysis

**Figure 4.**
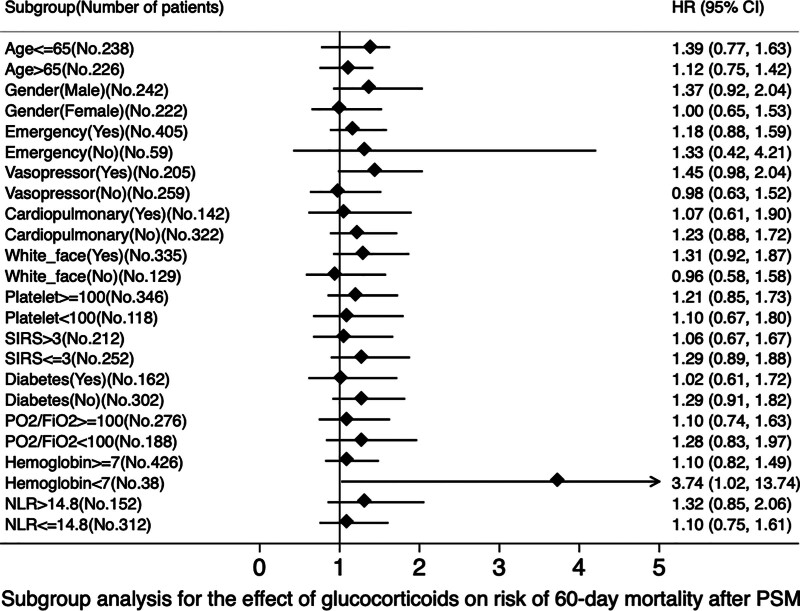
Subgroup analysis for the effect of glucocorticoids on risk of 60-day mortality after PSM. FiO2 = fraction of inspired oxygen, PO2 = Partial Pressure of Oxygen in Arterial Blood, NLR = neutrophil-to-lymphocyte ratio, PSM = propensity score matching, SIRS = systemic inflammatory response syndrome.

**Figure 5.**
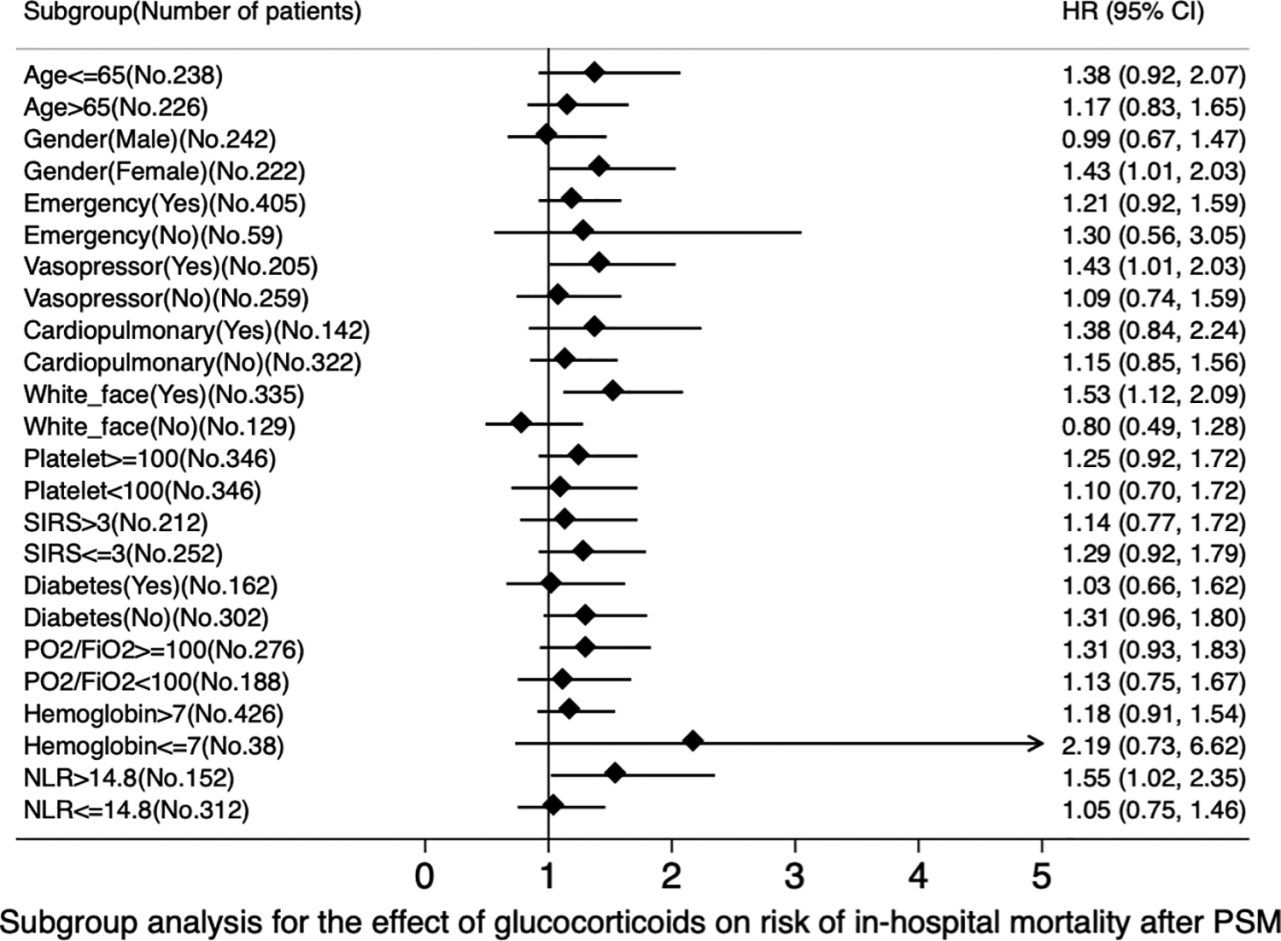
Subgroup analysis for the effect of glucocorticoids on risk of in-hospital mortality after PSM. FiO2 = fraction of inspired oxygen, PO2 = Partial Pressure of Oxygen in Arterial Blood, NLR = neutrophil-to-lymphocyte ratio, PSM = propensity score matching, SIRS = systemic inflammatory response syndrome.

The numbers of patients in each subgroup are shown in Figures 4, 5 and Tables S3, S4, http://links.lww.com/MD/N318. Interaction terms were tested and reported to assess the relationship between glucocorticoid treatment and 60-day mortality. The analysis indicated that glucocorticoids were associated with increased 60-day mortality in patients with hemoglobin levels below 7 g/dL or on vasoactive agents. The results also showed that glucocorticoids increased in-hospital mortality in male patients with ARDS (all *P* < .05; Fig. 4, Table S3, http://links.lww.com/MD/N318). Another subgroup of data showed that glucocorticoids were associated with increased in-hospital mortality in ARDS patients who were male, on vasoactive agents, Caucasians, or had a high NLR (neutrophil lymphocyte ratio > 14.8; all *P* < .05; Fig. 5, Table S4, http://links.lww.com/MD/N318).

## 10. Discussion

Our results demonstrate that glucocorticoid treatment significantly increased the 60-day mortality in ARDS patients. Additionally, glucocorticoid treatment increased in-hospital mortality and ICU stay length while reducing ventilator-free days at day 28. Although the impact on ICU mortality and 28-day mortality was not significant, there were clear negative effects on in-hospital and mid-term mortality.

The mechanisms behind the increased 60-day mortality with glucocorticoid treatment are multi-faceted. Previous meta-analyses have shown that corticosteroids may have a statistically insignificant improvement in short-term mortality in RCTs, but did not reduce long-term mortality in either RCTs or cohort studies.^[10]^ None of these results demonstrated a clear benefit of glucocorticoids. However, this study found that glucocorticoids significantly increased in-hospital mortality in patients with ARDS in the pre- and postmatch cohorts, whereas ICU mortality and 28-day mortality showed a nonsignificant worsening trend. The reasons for this difference are as follows: First, the severity of ARDS may explain the difference in the effects of glucocorticoid therapy. In the Meduri et al^[16]^ study, glucocorticoids improved survival only for severe ARDS, while only 33% of patients with severe ARDS were included in this study. However, our subgroup analysis did not show that severe ARDS improved ICU survival (HR, 1.32; 95% confidence interval 0.81–2.17; *P* = .270). Second, differences in inflammatory states due to the heterogeneity of etiology affect the efficacy of glucocorticoids. Lower respiratory tract transcriptional profiling of 52 patients with ARDS from COVID-19 or other etiologies showed that, in contrast to a cytokine storm associated with ARDS due to other causes, COVID-19 ARDS was characterized by a dysregulated host response, which is expected to be altered by dexamethasone.^[17]^ Our subgroup analysis by SIRS score did not show that glucocorticoids had an advantage in patients with ARDS with SIRS scores of 4. Instead, glucocorticoids increased the length of ICU stay and reduced ventilator-free days to 28 days. Third, different types of glucocorticoids may lead to different prognoses. However, subgroup analysis of methylprednisolone, dexamethasone, and hydrocortisone succinate did not show more beneficial for in-hospital survival. Of course, we did not exclude all factors, such as different glucocorticoid doses and timing of administration, which may also affect treatment outcome.^[7,10]^ For example, high doses of glucocorticoids may increase the risk of secondary infections, while moderate doses can offer benefits in persistent ARDS cases. Moreover, the timing of administration is crucial; early use (within 7–13 days of onset) may improve cardiopulmonary function, whereas late use (after 14 days) could lead to higher mortality rates due to advanced pulmonary fibrosis and inflammation.^[7]^ Early administration of dexamethasone could reduce the duration of mechanical ventilation and overall mortality in patients with moderate-to-severe ARDS.^[9]^ However, despite improvements in cardiopulmonary physiology, the routine use of methylprednisolone is not recommended for persistent ARDS. Additionally, starting methylprednisolone therapy more than 2 weeks after the onset of ARDS may increase the risk of death.^[7]^

The 60-day mortality is considered a reasonable endpoint for evaluating corticosteroid therapy for ARDS,^[18]^ as it avoids the observation that glucocorticoid therapy for ARDS may provide initial benefits by inhibiting inflammatory processes,^[19]^ while also taking into account that these benefits are quickly offset by delayed onset of adverse reactions, such as immunosuppression.^[20]^ This also reasonably explains the difference in survival between the early and late stages of ARDS caused by the use of glucocorticoids,^[21]^ and why the proportional hazards assumption test in Cox regression analysis in this study is not valid. We further performed time-stratified Cox analysis and found that glucocorticoids did not affect the mortality of ARDS from 0 to 14 days, but increased the mortality of ARDS from 15 to 60 days. The reasons for the survival of patients with glucocorticoid deterioration at later measurements (days 15–60) are as follows. First, glucocorticoid-induced immune dysfunction leads to ventilator-associated pneumonia (VAP), which is an independent predictor of death in patients with ARDS and is associated with prolonged MV duration and LOS.^[22]^ This is consistent with the results of this study in terms of longer ICU stay and a shorter duration of 28 days without ventilation. Studies have shown that patients with ARDS treated with dexamethasone developed VAP earlier, and monocyte human leukocyte antigen DR expression and circulating CD^4+^ cell counts were significantly reduced.^[13]^ NLR is an indicator of immune balance related to the prognosis of ARDS,^[23]^ and patients with high NLR (NLR ≥ 14.8) had shorter days free of mechanical ventilation and higher in-hospital and 30-day mortality. Cai et al^[24]^ found that corticosteroid therapy was associated with a lower risk of 60-day all-cause mortality in COVID-19 patients with a high NLR. However, the subgroup analysis in this study showed a high increase in in-hospital mortality from NLR in the glucocorticoid-treated group. This may be due to etiological heterogeneity, with a meta-analysis showing that glucocorticoids significantly increase mortality in influenza-associated ARDS,^[10]^ which may be attributed to prolonged viral shedding time and increased risk of superinfection.^[25,26]^ Second, the long-term side effects of glucocorticoids may be responsible for the lack of improvement the 60-day mortality. Glucocorticoids can affect neuromuscular function. Patients who survived ARDS had persistent functional limitations 1 year after discharge from the ICU, mainly due to muscle atrophy and weakness, and partly due to corticosteroid-induced and critical illness associated myopathy,^[27]^ which was associated with longer mechanical ventilation duration, ICU duration, and length of hospital stay.^[28,29]^ This disparity in glycemic control may also have contributed to the mortality and neuromuscular outcomes.^[7]^ The results of this study showed that the ventilator-free days on day 28 were significantly shortened in the glucocorticoid treatment group, indirectly suggesting that exposure to glucocorticoids may affect neuromuscular function and prevent improvement in mortality.

In addition to the above factors, cholesterol levels and glycated hemoglobin (HbA1c) levels may also serve as confounding variables affecting the efficacy of glucocorticoid treatment in ARDS. Elevated cholesterol levels, particularly the imbalance of low-density lipoprotein to high-density lipoprotein, can exacerbate lung injury by increasing pro-inflammatory cytokines and oxidative stress.^[30,31]^ Although glucocorticoids have anti-inflammatory effects, they may not sufficiently counteract the severe inflammatory state induced by high cholesterol. Additionally, elevated cholesterol can lead to endothelial dysfunction, further impairing vascular reactivity, worsening pulmonary edema and hypoxemia in ARDS patients, ultimately resulting in poor treatment outcomes. HbA1c levels reflect long-term blood glucose control and are crucial for ARDS prognosis. High HbA1c levels indicate chronic hyperglycemia, which impairs immune function and increases susceptibility to infections.^[32]^ In ARDS patients, this may lead to higher risks of VAP and other infections, complicating clinical management and increasing mortality.^[33]^ Moreover, hyperglycemia and insulin resistance, common in patients with high HbA1c, can be exacerbated by glucocorticoid therapy. Glucocorticoids can induce gluconeogenesis and inhibit insulin sensitivity, leading to steroid-induced diabetes. This hyperglycemic state not only increases infection risks but also promotes oxidative stress and inflammation, worsening lung injury and extending ICU stays.

Discussion or interpretation of subgroup analysis results. Wirtz et al^[34]^ found that monocyte IL-6 and TNF-alpha release was higher in male samples than in female samples, and glucocorticoid inhibition stimulated by lipopolysaccharide was less significant in male samples than in female samples. Wirtz et al’s^[34]^ research revealed that the release of monocyte IL-6 and TNF-alpha was elevated in male samples compared to female samples, while the inhibitory effect of lipopolysaccharide induced glucocorticoids was found to be less pronounced in male samples than in female samples. Monocytes in male ARDS patients may release more proinflammatory factors, which are less inhibited by glucocorticoids and may cause side effects, possibly responsible for the higher in-hospital mortality in male ARDS patients treated with glucocorticoids. While previous research has indicated that corticosteroids may have beneficial effects such as increasing ventilator-free days, improving oxygenation, and reducing vasopressor use,^[7,35]^ the current study revealed that glucocorticoids were associated with higher in-hospital mortality rates among ARDS patients receiving vasoactive medications. This may be due to steroid side effects, leading to a 34% increase in early infection complications and higher incidence of neuromuscular weakness incidence.^[7,36,37]^ The use of vasoactive agents in the ICU is more common in septic shock, which requires concurrent use of these agents to increase sensitivity. This also suggests that the degree of shock may be more severe in patients than in controls, explaining the increased in-hospital mortality caused by glucocorticoids. Glucocorticoid treatment increased the 60-day mortality in patients with hemoglobin levels below 7 g/dL. It is speculated that gastrointestinal bleeding cannot be ruled out due to decreased hemoglobin levels, and glucocorticoids may increase the risk of bleeding. In summary, our results do not support the use of glucocorticoids in ARDS patients.

This study has its limitations. First, this was a single-center retrospective cohort study; despite careful propensity score matching, residual confounding cannot be fully excluded. Second, changes in treatment strategies for critically ill patients, including metabolic and nutritional support strategies, mechanical ventilation methods, and the prone position, may influence the outcome of ARDS. Third, it is difficult to determine whether glucocorticoids were used for the anti-inflammatory treatment of ARDS, which may affect the results of the study. Fourth, only ARDS patients who used glucocorticoids after the onset of ARDS were included in our analysis; therefore, the effect of glucocorticoid prevention on the outcome of ARDS needs to be further studied. In summary, the results need to be verified in multi-center trials.

## 11. Conclusion

In critically ill patients with ARDS, glucocorticoid treatment increased in-hospital mortality, 15 to 60-day mortality, extended ICU stay, and reduced ventilator-free days at day 28 but did not significantly affect ICU mortality and 14-day and 28-day mortality. The overall results of this study do not support the use of glucocorticoid anti-inflammatory therapy in ARDS patients, particularly considering its adverse impact on in-hospital and mid-term mortality. Future research should focus on identifying patient subgroups who might benefit from glucocorticoid therapy and determining the optimal timing and dosing of treatment to minimize risks and enhance outcomes.

## Acknowledgments

We thank Dr Hui Chen from The Affiliated Zhongda Hospital of Southeast University, Dr Guoping Chen from the Anhui Provincial Center for Disease Control and Prevention, and Dr Wei Zhang from the Respiratory Department of Shaanxi Provincial People’s Hospital for their guidance in statistical analysis. We also thank the participants, developers, and investigators associated with the MIMIC-III database.

## Author contributions

**Conceptualization:** Zhonghua Lu, Yun Sun.

**Data curation:** Lijun Cao.

**Formal analysis:** Lijun Cao.

**Funding acquisition:** Zhonghua Lu.

**Investigation:** Yan Tang, Mei Liu, Hu Chen.

**Methodology:** Zhonghua Lu, Lijun Cao, Hu Chen.

**Project administration:** WeiLi Yu, Yun Sun.

**Resources:** WeiLi Yu.

**Software:** Zhonghua Lu, Yan Tang, Mei Liu, Lijun Cao.

**Supervision:** WeiLi Yu.

**Validation:** Yan Tang, Hu Chen.

**Visualization:** Mei Liu, Hu Chen.

**Writing—original draft:** Zhonghua Lu, Mei Liu.

**Writing—review & editing:** WeiLi Yu, Yun Sun.

## Supplementary Material
