# Supplementary material for: Association between glucocorticoid administration and outcomes in patients with ARDS based on the MIMIC-III database
Source: Medicine (Baltimore). 2024 Aug 9;103(32):e39239. doi: 10.1097/MD.0000000000039239 (PMC11315533; doi:10.1097/MD.0000000000039239)

| **Variables** | **HR** | **95% CI** | **P value** |
| --- | --- | --- | --- |
| Age | 1.008 | 1.02-1.56 | 0.033 |
| emergency | 1.798 | 1.35-2.39 | <0.001 |
| Ethnicity（white face） | 1.11 | 1.06-1.17 | <0.001 |
| Vasoactive treatment | 1.561 | 1.32-1.85 | <0.001 |
| ethnicity | 0.706 | 0.59-0.85 | <0.001 |
| cardiopulmonary | 0.765 | 0.62-0.94 | <0.001 |
| diabetes | 0.669 | 0.55-0.82 | <0.001 |
| CRRT | 1.28 | 1.00-1.64 | 0.047 |
| NLR | 1.01 | 1.00-1.01 | <0.001 |
| sapsii | 1.02 | 1.01-1.03 | <0.001 |
| BUN_max | 1.00 | 1.00-1.01 | 0.047 |

**Table S1 Potential risk variables for 60-day mortality**

***Abbreviations:*** *CRRT: Continuous Renal Replacement Therapy; NLR: Neutrophil-to-Lymphocyte Ratio; SAPSII: Simplified Acute Physiology Score II; BUN: Blood Urea Nitrogen; HR: Hazard Ratio; CI: Confidence Interval.*

**Table S2 Comparisons after propensity score matching for 60-day mortality.**

| Variables | **Non-GC（No. 232）** | **GC（No. 232）** | SMD-matched | **P value** |
| --- | --- | --- | --- | --- |
| Age (year) | 65.77(50.30, 76.38) | 62.56(51.02, 76.27) | 0.1 | 0.988 |
| Male | 117(50.43%) | 125 (53.88%) | 7.0 | 0.457 |
| Weight | 79.45(565.90, 94.75) | 78.05(67.00,90.85) | 3.6 | 0.678 |
| Height | 168.00(162.56,.177.80) | 172.72(162.56,182.88) | 14.0 | 0.111 |
| Emergency | 201(86.63%) | 204 (87.35%) | 3.6 | 0.676 |
| Ethnicity (White) | 167(71.98%) | 168 (72.41%) | 1.0 | 0.917 |
| SIRS | 3.00(3.00, 4.00) | 3.00 (3.00, 4.00) | 3.0 | 0.753 |
| WBC__Max_ | 14.85(10.20, 20.75) | 14.05 (10.25,20.05) | 7.4 | 0.384 |
| Plt__Min_ | 167.00(102.00, 262.50) | 178.50 (95.00,270.00) | 4.0 | 0.671 |
| Hemoglobin | 9.30(8.30, 10.75) | 9.40 (8.40,10.90) | 3.1 | 0.738 |
| Bun__Max_ | 29.50(17.00, 46.00) | 26.50 (18.00,41.50) | 0.3 | 0.975 |
| Lac__Max_ | 3.00(1.70, 6.55) | 2.80 (1.80,6.10) | 0.6 | 0.949 |
| NLR at diagnosis | 11.53(6.53, 17.15) | 11.26 (5.14,20.38) | 1.5 | 0.883 |
| Smoking | 174(75.00%) | 169 (73.88%) | 4.8 | 0.597 |
| SAPSII | 40.00(32.00, 53.00) | 43.00 (33.00,53.00) | 3.5 | 0.712 |
| Cardiopulmonary | 75(32.33%) | 67 (28.88%) | 7.6 | 0.420 |
| Diabetes | 81(34.91%) | 81 (34.91%) | 0.0 | 1.000 |
| PaO2/FiO2 at diagnosis | 116.61(77.50, 159.00) | 112.29 (79.50,168.00) | 0.0 | 0.998 |
| CRRT | 31(13.36%) | 29 (12.50%) | 2.8 | 0.782 |
| Vasoactive treatment | 101(43.53%) | 104 (44.83%) | 1.0 | 0.779 |

*Table S2 Data are presented as Number (%) or Median [interquartile range (IQR)]. Abbreviations: CRRT: Continuous Renal Replacement Therapy; NLR: Neutrophil-to-Lymphocyte Ratio; SAPSII: Simplified Acute Physiology Score II; PaO2: Partial Pressure of Oxygen; FiO2: Fraction of Inspired Oxygen; SIRS: Systemic Inflammatory Response Syndrome; WBC: White Blood Cell Count; Plt: Platelet Count; Bun: Blood Urea Nitrogen; Lac: Lactate; GC: Glucocorticoids; SMD: Standardized Mean Difference.*

**Table S3 Subgroup analysis for the effect of glucocorticoids on risk of 60-day mortality after PSM**

| **Subgroup** | No.of patients | *P* value | HR | Lower 95% CI | Upper 95% CI |
| --- | --- | --- | --- | --- | --- |
| Age |  |  |  |  |  |
| <=65 | 238 | 0.155 | 1.39 | 0.77 | 1.63 |
| >65 | 226 | 0.558 | 1.12 | 0.75 | 1.42 |
| emergency |  |  |  |  |  |
| Yes | 405 | 0.277 | 1.18 | 0.88 | 1.59 |
| No | 59 | 0.624 | 1.33 | 0.42 | 4.21 |
| Gender |  |  |  |  |  |
| Female | 222 | 0.998 | 1.00 | 0.65 | 1.53 |
| Male | 242 | 0.121 | 1.37 | 0.92 | 2.04 |
| Vasopressor therapy |  |  |  |  |  |
| Yes | 205 | 0.046 | 1.45 | 0.98 | 2.12 |
| No | 259 |  | 0.98 | 0.63 | 1.52 |
| cardiopulmonary |  |  |  |  |  |
| Yes | 142 | 0.809 | 1.07 | 0.61 | 1.90 |
| No | 322 | 0.237 | 1.23 | 0.88 | 1.72 |
| White face |  |  |  |  |  |
| Yes | 335 | 0.137 | 1.31 | 0.92 | 1.87 |
| No | 129 | 0.880 | 0.96 | 0.58 | 1.58 |
| platelet_min |  |  |  |  |  |
| >=100 | 346 | 0.290 | 1.21 | 0.85 | 1.73 |
| <100 | 118 | 0.714 | 1.10 | 0.67 | 1.80 |
| SIRS |  |  |  |  |  |
| >3 | 212 | 0.799 | 1.06 | 0.67 | 1.67 |
| <=3 | 252 | 0.085 | 1.29 | 0.89 | 1.88 |
| Diabetes |  |  |  |  |  |
| Yes | 162 | 0.941 | 1.02 | 0.61 | 1.72 |
| No | 302 | 0.158 | 1.29 | 0.91 | 1.82 |
| pao2fio2 |  |  |  |  |  |
| <100 | 188 | 0.263 | 1.28 | 0.83 | 1.97 |
| >=100 | 276 | 0.629 | 1.10 | 0.74 | 1.63 |
| hemoglobin_min |  |  |  |  |  |
| <7 | 38 | 0.047 | 3.74 | 1.02 | 13.74 |
| >=7 | 426 | 0.517 | 1.10 | 0.82 | 1.49 |
| NLR |  |  |  |  |  |
| <=14.8 | 312 | 0.638 | 1.10 | 0.75 | 1.61 |
| >14.8 | 152 | 0.213 | 1.32 | 0.85 | 2.06 |

*Abbreviations: PSM: Propensity Score Matching; HR: Hazard Ratio; CI: Confidence Interval; SIRS: Systemic Inflammatory Response Syndrome; PaO2: Partial Pressure of Oxygen; FiO2: Fraction of Inspired Oxygen; NLR: Neutrophil-to-Lymphocyte Ratio.*

**Table S4 Subgroup analysis for the effect of glucocorticoids on risk of in-hospital mortality after PSM**

| **Subgroup** | No.of patients | *P* value | HR | Lower 95% CI | Upper 95% CI |
| --- | --- | --- | --- | --- | --- |
| Age |  |  |  |  |  |
| <=65 | 238 | 0.122 | 1.38 | 0.92 | 2.07 |
| >65 | 226 | 0.834 | 1.17 | 0.83 | 1.65 |
| Gender |  |  |  |  |  |
| Female | 222 | 0.973 | 0.99 | 0.67 | 1.47 |
| Male | 242 | 0.042 | 1.43 | 1.01 | 2.03 |
| emergency |  |  |  |  |  |
| Yes | 405 | 0.165 | 1.21 | 0.92 | 1.59 |
| No | 59 | 0.543 | 1.30 | 0.56 | 3.05 |
| Vasopressor therapy |  |  |  |  |  |
| Yes | 205 | 0.046 | 1.43 | 1.01 | 2.03 |
| No | 259 | 0.676 | 1.09 | 0.74 | 1.59 |
| cardiopulmonary |  |  |  |  |  |
| Yes | 142 | 0.201 | 1.38 | 0.84 | 2.24 |
| No | 322 | 0.370 | 1.15 | 0.85 | 1.56 |
| White face |  |  |  |  |  |
| Yes | 335 | 0.008 | 1.53 | 1.12 | 2.09 |
| No | 129 | 0.345 | 0.80 | 0.49 | 1.28 |
| platelet_min |  |  |  |  |  |
| >=100 | 346 | 0.158 | 1.25 | 0.92 | 1.72 |
| <100 | 118 | 0.682 | 1.10 | 0.70 | 1.72 |
| SIRS |  |  |  |  |  |
| >3 | 212 | 0.528 | 1.14 | 0.77 | 1.72 |
| <=3 | 252 | 0.134 | 1.29 | 0.92 | 1.79 |
| Diabetes |  |  |  |  |  |
| Yes | 162 | 0.894 | 1.03 | 0.66 | 1.62 |
| No | 302 | 0.088 | 1.31 | 0.96 | 1.80 |
| PO2/FiO2 |  |  |  |  |  |
| <100 | 188 | 0.584 | 1.13 | 0.75 | 1.67 |
| >=100 | 276 | 0.121 | 1.31 | 0.93 | 1.83 |
| hemoglobin_min |  |  |  |  |  |
| <7 | 38 | 0.164 | 2.19 | 0.73 | 6.62 |
| >=7 | 426 | 0.216 | 1.18 | 0.91 | 1.54 |
| NLR |  |  |  |  |  |
| <=14.8 | 312 | 0.769 | 1.05 | 0.75 | 1.46 |
| >14.8 | 152 | 0.04 | 1.55 | 1.02 | 2.35 |

*Abbreviations: PSM: Propensity Score Matching; HR: Hazard Ratio; CI: Confidence Interval; SIRS: Systemic Inflammatory Response Syndrome; PaO2: Partial Pressure of Oxygen; FiO2: Fraction of Inspired Oxygen; NLR: Neutrophil-to-Lymphocyte Ratio.*


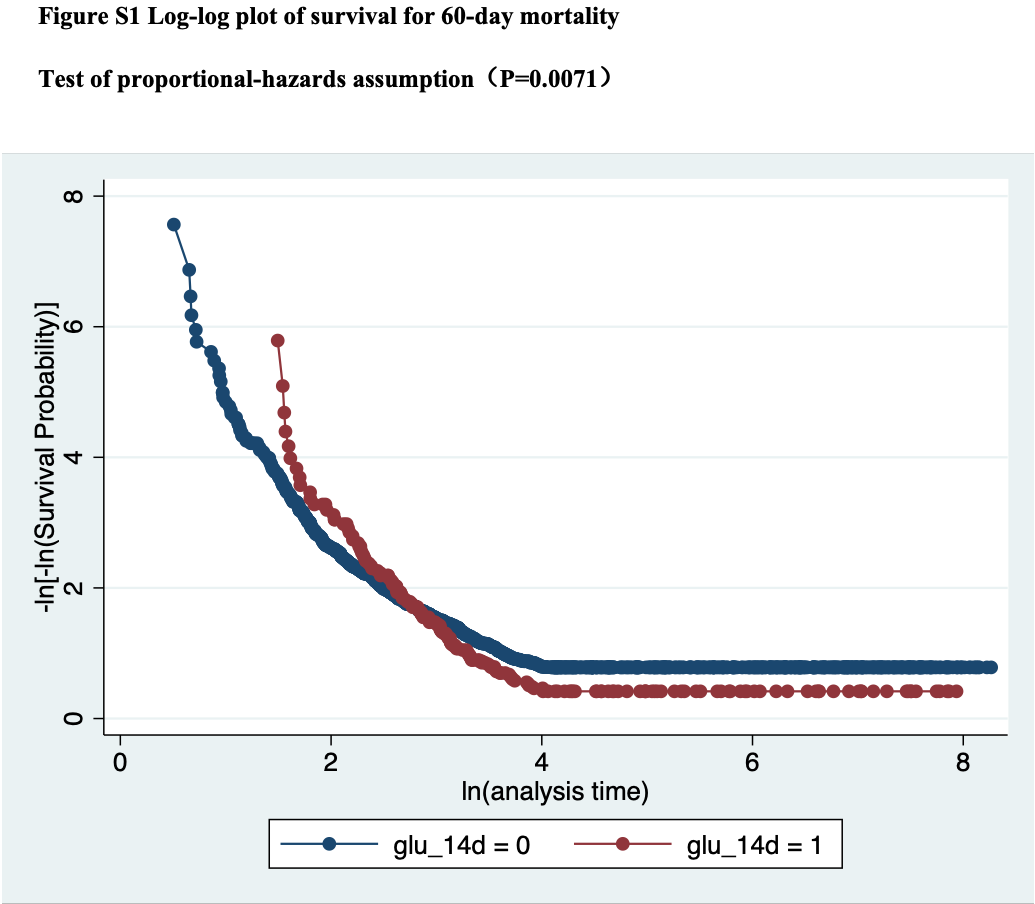


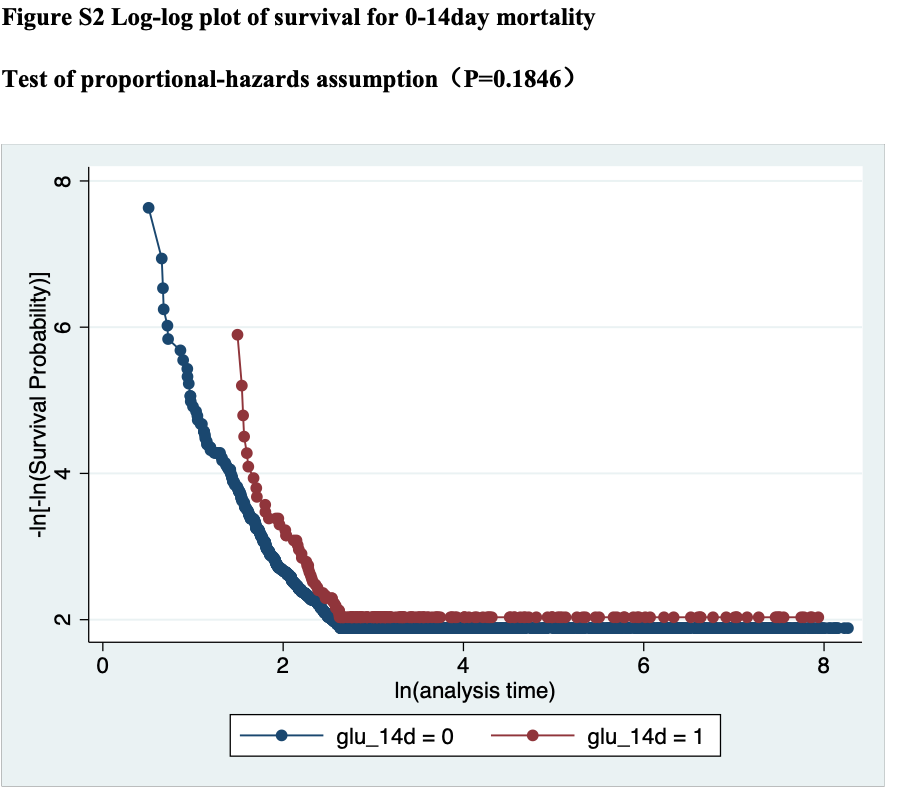


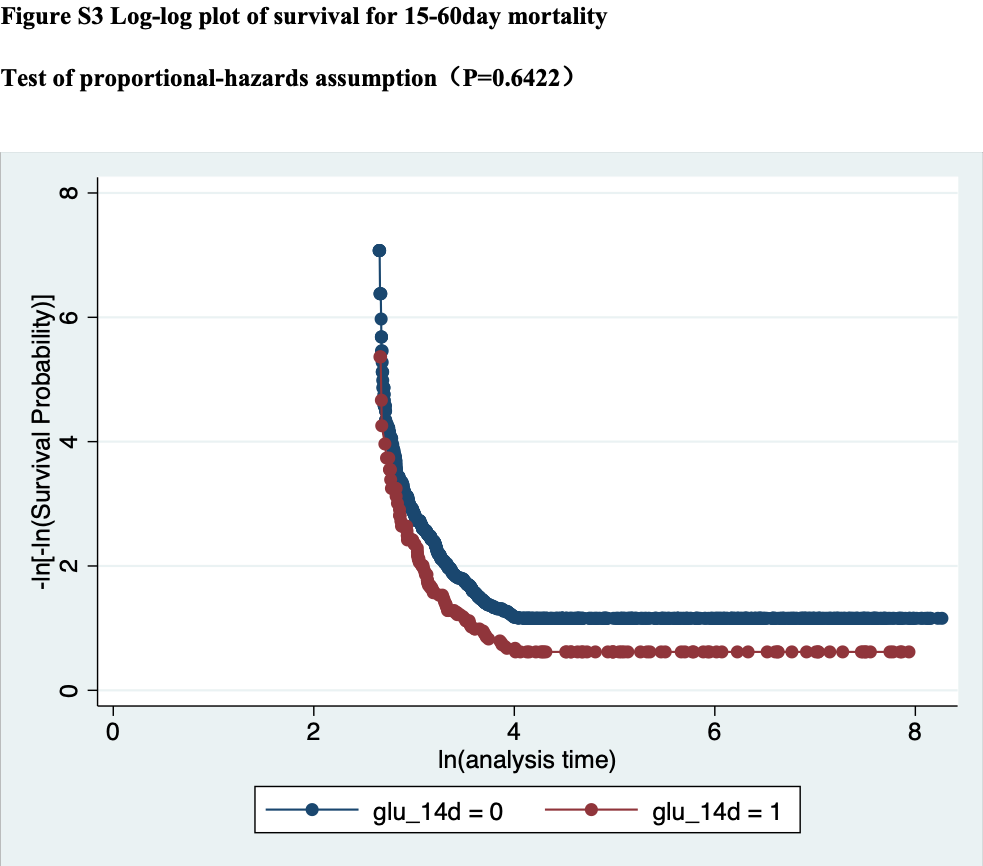

Supplement: Supplementary file 1 [file medi-103-e39239-s001.docx]
